# Risk factors for drug-related impaired gastric emptying: a pharmacovigilance analysis of the FDA adverse event reporting system

**DOI:** 10.3389/fphar.2026.1844057

**Published:** 2026-05-18

**Authors:** Jie-Hai Chen, Jia-Cong Chen, Ya-Jing Mei, Xiao-Chun Zhu, Qing-Ming Luo, Yuan-Yan Tu

**Affiliations:** 1 Department of Anesthesiology, Dongguan Maternal and Child Healthcare Hospital, Dongguan, Guangdong, China; 2 Division of Cardiology, The Affiliated Dongguan Songshan Lake Central Hospital, Guangdong Medical University, Dongguan, Guangdong, China; 3 Dongguan Maternal and Child Health Care Hospital, Dongguan, Guangdong, China

**Keywords:** adverse drug events, disproportionality analysis, GLP-1 receptor agonists, impaired gastric emptying, pharmacovigilance

## Abstract

**Background:**

Pulmonary aspiration remains a major perioperative patient safety issue. Drug-related impaired gastric emptying (IGE) is a recognized yet underappreciated risk factor for aspiration. With the increasing use of medications such as glucagon-like peptide-1 receptor agonists (GLP-1RAs), concerns have grown regarding their potential to delay gastric emptying and thereby elevate aspiration risk. However, real-world data on drug-related IGE remain limited.

**Objective:**

To identify risk factors for drug-related IGE using real-world pharmacovigilance data from the FDA Adverse Event Reporting System (FAERS).

**Methods:**

A retrospective pharmacovigilance study was conducted using FAERS data from Q1 2004 through Q2 2025. Disproportionality analysis, logistic regression, LASSO regression and time-to-onset analysis were performed to investigate the association between drugs and IGE from different perspectives.

**Results:**

A total of 731 drugs related to IGE. Six of the top 10 most frequently reported drugs associated with IGE were antidiabetic agents, including five GLP-1RAs: semaglutide, dulaglutide, tirzepatide, exenatide, and liraglutide. Multi-factor analysis identified female sex, age <63 years, and exposure to any of nine specific drugs—including GLP-1RAs, insulin lispro, sodium oxybate, olmesartan, and esomeprazole—as independent risk factors for drug-related IGE. The model achieved an ROC-AUC of 0.739, indicating good discriminatory capability. Time-to-onset analysis revealed an early-failure pattern for all nine drugs.

**Conclusion:**

Our study shows that female sex, younger age, and use of specific medications are associated with an increased risk of drug-related IGE. These findings may provide valuable evidence to support the early recognition of drug-related IGE and for optimizing anaesthetic plans and perioperative personalized fasting strategies.

## 1 Introduction

Perioperative pulmonary aspiration of gastric contents remains a critical threat to patient safety in contemporary anesthetic practice, and its management is frequently judged to be substandard (Warner et al., 2021). Identified risk factors for pulmonary aspiration include emergency procedures, gastrointestinal obstruction, recent opioid administration, treatment for diabetes (Warner et al., 2021). Drug-related factors are recognized as an etiology of perioperative pulmonary aspiration and impaired gastric emptying (IGE) (Warner et al., 2021; El Halabi and Parkman, 2023). IGE is a clinical condition characterized by a delay in the transit of gastric contents into the duodenum. The clinical implications of IGE extend beyond gastrointestinal discomfort. Critically, IGE increases residual gastric volume, thereby elevating the risk of pulmonary aspiration of gastric contents during induction of general anesthesia, procedural sedation, or in emergency airway management scenarios. Aspiration events are associated with significant morbidity, including aspiration pneumonitis, acute respiratory distress syndrome, and increased mortality.

Drug-related IGE, or drug-related gastroparesis, has been increasingly encountered in the clinical practice of physicians and has attracted the attention of researchers. The use of opioid medications is associated with an increased severity of gastric emptying delay, with half of opioid users experiencing severely delayed gastric emptying (Navas et al., 2021). In addition to opioid analgesics, other medications associated with IGE include glucagon-like peptide-1 receptor agonists (GLP-1RAs), anticholinergic drugs, tricyclic antidepressants, calcium channel blockers, and cyclosporine (Ye et al., 2022). Driven by the rapid increase in GLP-1RAs prescriptions, their well-documented delayed gastric emptying has heightened perioperative concern regarding an elevated risk of pulmonary aspiration under general anesthesia (Van Zuylen et al., 2024). However, a recently published systematic review analyzed randomized trials examining the effects of GLP-1RAs on gastric emptying in obese populations (Aldawsari et al., 2023). Two studies employing semaglutide failed to observe significant delayed gastric emptying, whereas two liraglutide trials consistently reported delays. Inconsistent findings regarding IGE, combined with its significant burden on healthcare systems and patients (Ye et al., 2022), highlight a critical knowledge gap. Compounding this issue, anesthesiologists often have only a fragmented understanding of drug-related IGE, leading to underappreciation of key perioperative risk factors (Warner et al., 2021). Therefore, real-world evidence from large pharmacovigilance databases is urgently needed to delineate the spectrum of drug-related IGE, enabling anesthesia providers to better recognize and mitigate potentially catastrophic aspiration events.

This study aimed to conduct a comprehensive evaluation of risk factors associated with drug-related IGE. Given that the US Food and Drug Administration (FDA) Adverse Event Reporting System (FAERS) database is currently the largest adverse event (AE) reporting repository, we employed this database to perform a comprehensive investigation into drug-related IGE and identify potential risk factors. The findings were anticipated to furnish pivotal evidence for future clinical investigations and to enhance patient safety through more informed perioperative management.

## 2 Materials and methods

### 2.1 Data source

We conducted a retrospective pharmacovigilance study spanning Q1 2004 through Q2 2025 using the publicly available FAERS database (https://fis.fda.gov/extensions/FPD-QDE-FAERS/FPD-QDE-FAERS.html). The voluntary reporting system compiles spontaneous AE reports from healthcare professionals and consumers. Each file contained seven standard domains: DEMO (patient demographic and administrative information), DRUG (suspect/concomitant medicines), REAC (adverse drug reactions), OUTC (patient outcomes), THER (therapy dates), RPSR (report sources) and INDI (indications). To eliminate redundant records, we followed FDA-recommended deduplication logic: report was sorted in sequence according to CASEID, FDA_DT and PRIMARYID. For identical CASEIDs, the report with the latest FDA_DT was retained; when both CASEID and FDA_DT were identical we preserved the highest PRIMARYID, and further duplicate PRIMARYID entries were deleted to ensure each AE report was represented only once in the analytic set. IGE cases were ascertained by applying the Medical Dictionary for Regulatory Activities (MedDRA) version 28.0, restricting the cohort to reports mapped to the MedDRA Preferred Term “impaired gastric emptying”. Reports in which a drug was designated as the “primary suspect” for IGE constituted our analytic cohort.

### 2.2 Disproportionality analysis

In pharmacovigilance research, disproportionality analyses were chiefly employed to evaluate potential associations between individual drugs and specific AEs. We mined safety signals with four algorithms: Reporting Odds Ratio (ROR) (Rothman et al., 2004), Proportional Reporting Ratio (PRR) (Evans et al., 2001) with χ^2^ testing, Multi-item Gamma-Poisson Shrinker (MGPS) (Heo and Jung, 2020), and Bayesian Confidence Propagation Neural Network (BCPNN) for Information Component (IC) estimation (Bate, 2007), thereby enhancing the robustness of detected safety signals. All four algorithms are based on 2 × 2 contingency tables (Supplementary Table S1). Computational expressions and signaling thresholds for all four disproportionality algorithms are detailed in Supplementary Table S2.

### 2.3 Regression analysis

To delineate independent predictors of drug-related IGE, we extracted FAERS records with complete patient-level covariates (sex, age, weight) and implemented a three-step modelling strategy. First, we performed single-factor analysis in which a drug was considered a suspect if it generated positive signals across all four disproportionality metrics (ROR, PRR, MGPS, and BCPNN), a > 100 reports, and survived Bonferroni correction (adjusted p < 0.01). Second, Drugs with p < 0.01 in these univariable models were advanced to least absolute shrinkage and selection operator (LASSO) regression to mitigate multicollinearity and overfitting. LASSO regression was performed using 10-fold cross-validation, and the penalty parameter (λ) that minimized the cross-validation binomial deviance (lambda.min) was selected as the final model, representing the optimal balance between fit and complexity. Only drugs retaining non-zero coefficients were retained. Finally, we constructed a multi-factor logistic regression model that incorporated the LASSO-selected drugs together with patient-level covariates (sex, age, weight) as independent variables to determine the existence of drug-related IGE risk factors.

### 2.4 Time-to-onset analysis and cumulative incidence

To characterize the temporal profile of drug-related IGE, we performed a time-to-onset analysis based on the Weibull shape parameter test (Sauzet et al., 2013)—an established pharmacovigilance tool that discriminates early-onset, constant, or late-rising hazards without requiring an external comparator cohort. All reports with missing therapy-start or AE-onset dates were excluded; when both dates were identical the latency was set to 1 day. Time-to-onset distributions were summarized by the median and interquartile range, and the Weibull parameters α (scale) and β (shape) were then estimated, focusing on their joint depiction of the central tendency and hazard evolution. Interpretation followed standard failure-time nomenclature: β < 1 with an upper 95% CI also below one implies a decreasing hazard (early failure pattern); β ≈ 1 with a 95% CI spanning 1 indicates a time-constant hazard (random failure pattern); β > 1 with a lower 95% CI above 1 reflects an increasing hazard (wear out pattern). Cumulative incidence of IGE were visualized with Kaplan-Meier curves, and inter-drug differences were evaluated by the log-rank test.

### 2.5 Statistical analysis

We used descriptive statistics to profile the clinical features of patients with drug-associated IGE reports. Bonferroni adjustment for multiple comparisons was applied to in the initial disproportionality analysis and multivariable logistic regression. The threshold for significance was set at a Bonferroni-corrected p-value (p-adjust) of <0.01. The Mann-Whitney U test was employed to compare differences in the onset times of various drugs associated with IGE. This study was conducted based on the reporting of a disproportionality analysis for drug safety signal detection using individual case safety reports in pharmacovigilance (READUS-PV) (Fusaroli et al., 2024) (see Supplementary Table S3). All Data processing and statistical analysis were carried out in R version 4.3.3.

## 3 Results

### 3.1 Baseline characteristics of IGE

**FIGURE 1:**
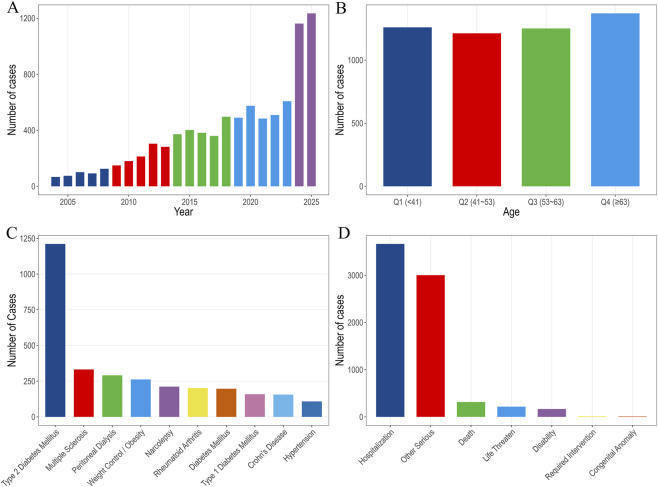
Relevant information from various drug-related IGE reports. **(A)** Drug-related IGE reports by year; **(B)** Reporting rate of drug-related IGE by patient age; **(C)** Diagnosis of patients with drug-related IGE; **(D)** Adverse reactions’ outcome with drug-related IGE. IGE, impaired gastric emptying.

Figure 1 depicts the baseline characteristics of drug-related IGE. FAERS database reports spanning Q1 2004 to Q2 2025 were analyzed. After deduplication, a total of 18,937,868 AE reports were identified, of which 8,664 were related to IGE. Females accounted for the majority of reports (5,674; 65.5%), males for 2,045 (23.6%), and gender information was missing for 945 reports (10.9%). Median age was 53 years (interquartile range [IQR] 41–63 years) and median body weight 78 kg (IQR 64–95.2 kg). Data from Figure 1A suggest a rising trend in the number of reports associated with drug-related IGE, with a marked increase observed in the most recent years (2024–2025), indicating an accelerating growth rate in these years. Regarding age distribution (Figure 1B), the reports were fairly evenly spread across different age groups, although the incidence of drug-related IGE was notably higher among patients aged 63 years and older. Figure 1C highlights diabetes as the most common diagnosis linked to IGE cases, while Figure 1D shows that the primary adverse consequence was extended hospitalization.

### 3.2 Drugs associated with IGE

**FIGURE 2:**
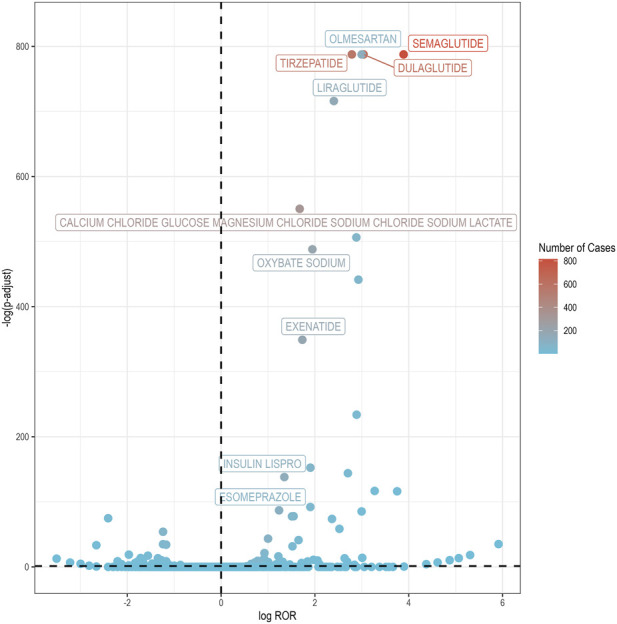
IGE-related drug volcano plots. IGE, impaired gastric emptying; ROR, reporting odds ratio; p-adjust, p-value after Bonferroni correction. The x-axis displays the logarithm of the ROR, wherein positive displacements correspond to an elevated frequency of adverse event reports relative to other adverse events. The y-axis shows the negative logarithm of the Bonferroni-corrected p-value derived from Fisher’s exact test, where greater heights reflect stronger statistical significance. Color intensity encodes report counts on a red gradient, with deeper saturation representing higher frequencies. Agents in the upper-right quadrant concurrently exhibit large effect sizes (ROR) and high statistical significance (p-adjust), suggesting the most robust signals for IGE.

Volcano plots were used to explore the association between IGE and suspect medications (Figure 2). The horizontal axis represents the logarithm of the ROR, with positive values suggesting that the adverse effects of drug-related IGE were more frequent relative to other adverse effects. The vertical axis corresponds to the negative logarithm of the adjusted p-value, derived from the p-value after Fisher’s exact test and Bonferroni correction, with higher values indicating a more significant statistical difference. The dot coloration corresponds to the reported cases, where a more intense red hue signifies a greater number of case reports. Consequently, medications positioned in the upper right quadrant of the plot exhibit both a substantial signal strength and a significant difference.

Among 731 agents linked to IGE, 42 achieved positive safety signals across all four disproportionality metrics (ROR, PRR, MGPS, and BCPNN), as detailed in Supplementary Table S2. Table 1 lists the top 10 reported drugs associated with IGE incident. It is noteworthy that six of these top drugs are categorized as antidiabetic medications, with five specifically falling under the GLP-1RAs subgroup, namely, semaglutide, dulaglutide, tirzepatide, exenatide, and liraglutide. The remaining drugs span various therapeutic classes, including nutritional supplements, narcolepsy treatments, antihypertensive agents, and gastric acid secretion inhibitors.

**TABLE 1:** The signal strength of top 10 drugs related to IGE.

Drug	IGE cases	ROR (95% CI)	PRR (95% CI)	χ2	EBGM (EBGM05)	IC (IC025)
Semaglutide	815	48.93 (45.5–52.63)	47.99 (44.68–51.55)	33,990.04	43.57 (40.52)	5.45 (5.27)
Dulaglutide	601	20.85 (19.18–22.66)	20.68 (19.04–22.45)	10,477.16	19.31 (17.77)	4.27 (4.11)
Tirzepatide	559	16.23 (14.89–17.68)	16.12 (14.8–17.56)	7,421.20	15.15 (13.9)	3.92 (3.76)
Calcium chloride/glucose/magnesium chloride/sodium chloride/sodium lactate*	328	5.34 (4.78–5.96)	5.33 (4.77–5.95)	1,110.46	5.17 (4.63)	2.37 (2.19)
Oxybate sodium	197	7.00 (6.08–8.07)	6.99 (6.07–8.04)	987.82	6.85 (5.95)	2.78 (2.53)
Exenatide	190	5.65 (4.89–6.52)	5.63 (4.88–6.51)	708.80	5.53 (4.79)	2.47 (2.22)
Liraglutide	162	11.06 (9.46–12.92)	11.01 (9.43–12.86)	1,447.22	10.82 (9.26)	3.44 (3.12)
Insulin lispro	138	3.85 (3.25–4.56)	3.85 (3.25–4.55)	286.05	3.80 (3.21)	1.93 (1.65)
Olmesartan	124	20.01 (16.75–23.91)	19.84 (16.63–23.68)	2,188.25	19.58 (16.38)	4.29 (3.83)
Esomeprazole	108	3.44 (2.84–4.15)	3.43 (2.84–4.15)	183.86	3.4 (2.81)	1.77 (1.46)

IGE, impaired gastric empty; ROR, reporting odds ratio; CI, confidence interval; PRR, proportional reporting ratio; χ2, chi-squared; IC, information component; IC025, the lower limit of the 95% one-sided CI of the IC; EBGM: empirical bayes geometric mean; EBGM05, The lower limit of the 90% one-sided CI of the EBGM.

* represents a peritoneal dialysis solutions.

In the FAERS database, AE reports linked to the top 10 drugs documented 3,222 cases of IGE and 348 cases of pulmonary aspiration (Supplementary Table S4); the latter corresponds to 10.8% of the former reports. Among the pulmonary aspiration-related reports associated with these 10 drugs, only esomeprazole generated a consistent safety signal across all four disproportionality algorithms; semaglutide met the criteria for three, and sodium oxybate for two, whereas no quantitative signal was detected for the remaining agents (Supplementary Table S4).

### 3.3 Risk factors for drug-related IGE

**FIGURE 3:**
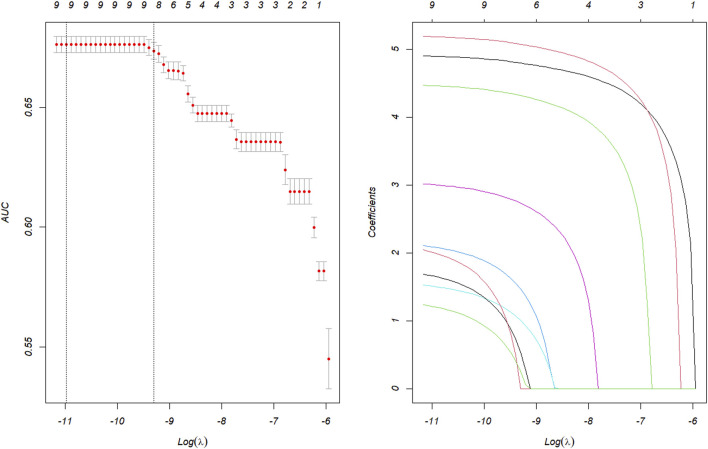
Results of the LASSO regression analysis. LASSO, least absolute shrinkage and selection operator.

**FIGURE 4:**
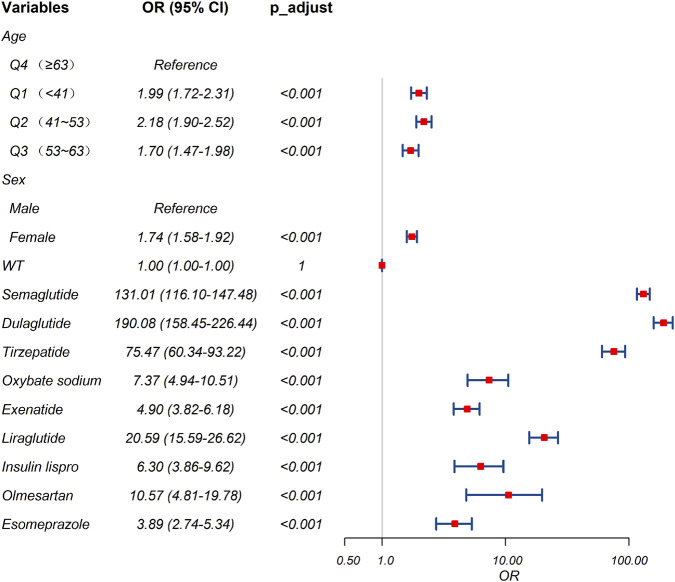
Results of the multi-factor logistic regression analysis. CI, confidence interval; OR, odds ratio; p_adjust, p-value after Bonferroni correction. A p_adjust value <0.01 indicates statistical signiffcance.

**FIGURE 5:**
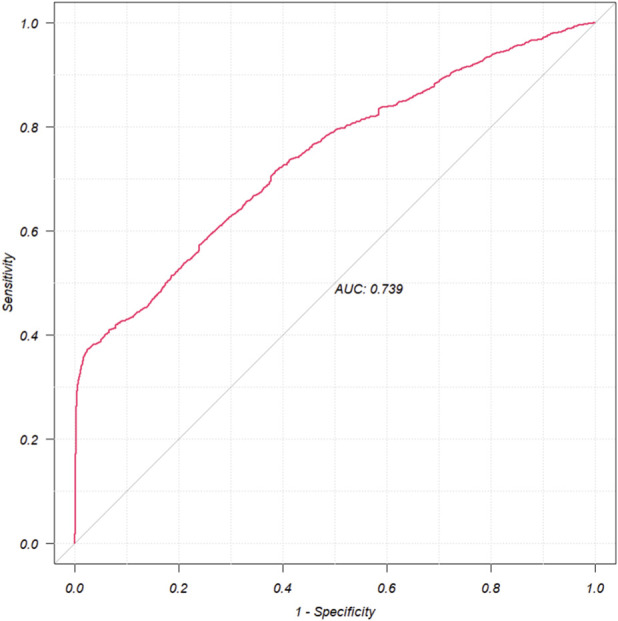
The ROC curves of drug-related IGE risk factors. IGE, impaired gastric emptying; ROC, receiver operating characteristic; AUC, area under curve.

A total of 10 suspected drugs (Table 1) met all four algorithmic thresholds, exceeded 100 case reports, and achieved a Bonferroni-adjusted p-value <0.01. Each of these drugs was subsequently evaluated using single-factor logistic regression models. Drugs with p < 0.01 in single-factor analysis were advanced to LASSO regression, yielding nine candidates (Figure 3). These nine drugs, together with patient demographics, were entered into a multivariable logistic model to quantify independent IGE risk (Figure 4). After adjusting for drug exposure and other covariates, multi-factor analysis identified female sex, age younger than 63 years and exposure to any of nine agents including semaglutide, dulaglutide, tirzepatide, sodium oxybate, exenatide, liraglutide, insulin lispro, olmesartan or esomeprazole as independent risk factors of drug-related IGE. The model achieved a discriminative performance with an ROC-AUC of 0.739 (Figure 5).

### 3.4 Time-to-onset analysis and cumulative incidence

**FIGURE 6:**
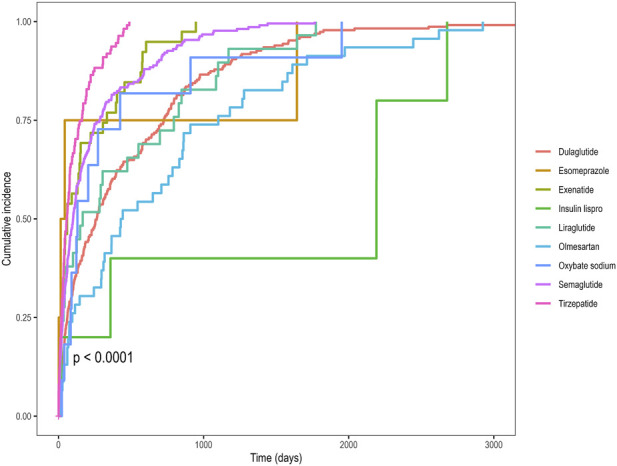
Comparative analysis of cumulative incidence of IGE events in patients receiving treatment with the nine drugs identified by LASSO regression. IGE, impaired gastric emptying; LASSO, least absolute shrinkage and selection operator.

Table 2 summarizes the time-to-onset characteristics for the nine drugs retained in the LASSO regression model, showing median onset times ranging from 21.5 to 1275.5 days. All the nine drugs displayed a consistent early-failure pattern, with the hazard of IGE declining over time following drug initiation (Table 2). Figure 6 shows Kaplan-Meier curves for IGE time to onset. The log-rank test revealed significant differences across the drugs (p < 0.0001; Figure 6). Supplementary Figure S1 illustrates the distribution of time-to-onset for drugs. Notably, heterogeneity in time-to-onset was also observed within the GLP-1RAs class. Among GLP-1RAs, semaglutide, tirzepatide, exenatide and liraglutide showed no significant pairwise differences, whereas dulaglutide had a markedly longer onset interval than any of these four (Supplementary Figure S1).

**TABLE 2:** Time-to-onset analysis limited to the nine IGE-associated drugs identified by LASSO regression.

Drug	Cases (n)	Weibull distribution					Failure type
		Time-to-onset (days)	Scale parameter		Shape parameter		
		Median (IQR)	α	95% CI	β	95% CI	
Semaglutide	815	42.5 (1.0–176.8)	80.17	60.4–99.94	0.48	0.44–0.53	Early failure
Dulaglutide	601	243.5 (49.0–703.0)	360.05	291.36–428.75	0.69	0.62–0.76	Early failure
Tirzepatide	559	40.5 (6.8–121.2)	66.09	47.46–84.73	0.64	0.55–0.72	Early failure
Oxybate sodium	197	88.0 (1.0–222.8)	122.46	−14.28–259.2	0.46	0.28–0.64	Early failure
Exenatide	190	21.5 (1.0–137.2)	51.56	21.43–81.68	0.45	0.36–0.54	Early failure
Liraglutide	162	46.0 (2.0–513.5)	146.99	37.36–256.63	0.44	0.33–0.56	Early failure
Insulin lispro	138	1,275.5 (105.5–2,192.8)	799.39	−544.11–2,142.89	0.50	0.15–0.85	Early failure
Olmesartan	124	426.0 (75.0–910.0)	567.49	333.99–801	0.71	0.55–0.88	Early failure
Esomeprazole	108	30.0 (12.8–444.0)	145.09	−209.05–499.23	0.43	0.11–0.74	Early failure

IGE, impaired gastric empty; LASSO, least absolute shrinkage and selection operator; n, number of cases with available time-to-onset; IQR, interquartile range; CI, confidence interval. Weibull distribution parameters: α (scale parameter) reflects the central tendency of the time-to-onset distribution; β (shape parameter) characterizes the hazard function over time. β < 1 with an upper 95% CI < 1 indicates a decreasing hazard rate (“Early failure” pattern).

## 4 Discussion

Drug-induced is one of the most common etiologies of IGE (El Halabi and Parkman, 2023; Ye et al., 2022). Drugs that delay gastric emptying, such as opioids and the increasingly prevalent GLP-1RAs, are raising growing perioperative concerns due to their potential to increase the risk of pulmonary aspiration during general anaesthesia (Van Zuylen et al., 2024; Paggers et al., 2025). Comprehensive pharmacovigilance analyses are essential to elucidate the association between drugs and IGE, identify high-risk populations, and delineate the onset profiles of drug-related IGE.

Using data from the FAERS database, this study revealed the top ten drugs most frequently reported in association with IGE. Six of these were antidiabetic agents, five of which were GLP-1RAs, namely, semaglutide, dulaglutide, tirzepatide, exenatide, and liraglutide. Collectively, these five GLP-1RAs accounted for 72.2% (2,327/3,222) of IGE cases attributed to the top ten drugs, yet comprised only 26.9% (2,327/8,664) of all IGE reports in the database. While this indicates a disproportionately high reporting rate of IGE events associated with GLP-1RAs among the most frequently implicated drugs, their overall contribution to total IGE cases remains modest. These findings underscore the importance of heightened clinical awareness regarding potential IGE risks related to diabetes therapies, particularly GLP-1RAs, during perioperative management.

Our analysis revealed that drug-related IGE reports were more prevalent among female patients and in the age group older than 63 years. Further multivariate analysis indicated that female sex and younger age (<63 years) were independent risk factors for drug-related IGE. Our findings aligned with a previous report on GLP-1RAs associated-IGE events (Huang et al., 2025), collectively indicating that female sex and younger age confer a higher risk of IGE during treatment with specific drugs. The higher reported incidence of drug-related IGE among patients aged ≥63 years likely reflects the greater baseline medication use and comorbidity burden in older populations, which increases both drug exposure and opportunities for AEs reporting. These two observations are complementary. The unadjusted reporting patterns describe the overall case distribution, whereas the adjusted analysis identifies intrinsic risk. Specifically, after controlling for the significant effects of specific drugs (e.g., GLP-1RAs), younger age (<63 years) emerged as an independent risk factor. This indicates that for an equivalent exposure to a high-risk drug, younger patients may bear a disproportionately higher relative risk of developing IGE. A large-scale study of the US population aimed to assess the prevalence of gastroparesis, characterized by delayed gastric emptying (Ye et al., 2022). Similar to our findings, it revealed that the prevalence in female was more than twice that in male individuals. Sex hormones may account for this female predominance of gastroparesis (Dilmaghani et al., 2023), and age-related changes in gastrointestinal function may also play a role (Merchant et al., 2016). However, the biological mechanisms underlying these sex- and age-related differences remain elusive. Future studies should extensively investigate these mechanisms. When performing preoperative risk evaluations for patients undergoing treatment with these specific drugs with known pro-ileus potential, it is prudent to incorporate factors such as age and sex, as these may affect the susceptibility to IGE. Such a strategy could refine perioperative treatment regimens and individualize care.

A comprehensive multi-factor analysis of FAERS data identified nine agents as potential risk factors for drug-related IGE (Figure 4). Antidiabetic medications, particularly GLP-1RAs, topped the list, followed by narcolepsy treatments, antihypertensive agents, and gastric acid secretion inhibitors. Previous studies have identified diabetes and GLP1-RAs as risk factors for IGE (Huang et al., 2025; Al-Mulhim, 2017; Sen et al., 2024). In line with these reports, our study identified antidiabetic drugs GLP-1RAs (semaglutide, dulaglutide, tirzepatide, exenatide, and liraglutide) and insulin lispro were associated with an increased risk of IGE. Our analysis of the temporal distribution of drug-related IGE events revealed that the median time to onset varied considerably, ranging from 21.5 days (exenatide) to 1275.5 days (insulin lispro). Weibull shape parameter analysis indicated that IGE events associated with all nine drugs followed an “early failure” pattern, suggesting that the hazard rapidly decreases over time. Our findings aligned with prior reports showing that impaired gastric emptying and gastrointestinal adverse events associated with GLP-1RAs gradually diminished over time (Huang et al., 2025; Horowitz et al., 2017). The onset of GLP-1RAs-associated IGE did not differ among semaglutide, tirzepatide, exenatide, or liraglutide; in contrast, dulaglutide exhibited a significantly delayed onset compared with each of these four. This suggests that adopting a standardized approach to preoperative different GLP-1RA management warrants further consideration. Few studies reported associations between sodium oxybate, olmesartan, and esomeprazole with IGE. Mechanistic studies are warranted to clarify the causal pathways linking these agents to IGE. For patients taking these medications, preoperative care requires careful consideration.

This study has several strengths. Firstly, we conducted a comprehensive analysis of real-world data from the FAERS database, which provides a large sample size and extensive coverage of drug-related IGE adverse events. Secondly, we employed multiple statistical methods—including descriptive analysis, disproportionality analysis, logistic regression analysis, LASSO regression, and time-on-onset analysis—to explore the association between suspected drugs and IGE from multiple dimensions. This enabled the identification of risk factors and temporal patterns of occurrence. These findings may yield actionable hypotheses for future trials on fasting regimens and preoperative personalized risk assessment strategies.

Limitations inherent to the FAERS database inevitably constrain our study. First, spontaneous reporting is vulnerable to underreporting, duplicate entries and inaccurate reporting, all of which can bias effect estimates. Furthermore, interpretation of the FAERS data must account for notoriety bias (stimulated reporting). The marked increase in IGE reports observed in 2024–2025 coincides with heightened clinical awareness and numerous publications regarding perioperative aspiration risk with GLP-1RAs. This likely contributed to enhanced reporting vigilance, artificially amplifying the signal magnitude relative to other, less publicized drugs. Second, although we explored age, weight and sex as risk factors of drug-related IGE, the absence of data on race, co-morbidities, concomitant medications and dose precludes full adjustment for confounding. Crucially, confounding by indication is a major concern for antidiabetic agents. Diabetic gastroparesis, driven by autonomic neuropathy, is an established complication of diabetes. As FAERS lacks data on glycemic control, disease duration, or autonomic function, the IGE signals observed for GLP-1RAs may reflect the underlying disease burden and its complications rather than a pure pharmacological effect. The lack of standardized dosing information also prevents assessment of a dose-response relationship, which is a cornerstone of causality assessment in pharmacovigilance. Therefore, the strong signals observed for this drug class should not be interpreted in isolation from the underlying disease pathology. Third, disproportionality analysis generate hypotheses, not evidence of causality; thus the observed IGE signals require prospective verification. Fourth, FAERS does not capture the precise timing or clinical context of events, so we cannot determine what proportion of IGE reports occurred perioperatively or under anaesthesia. Fifth, time-to-onset analysis was restricted to reports with complete date information. Exclusion of reports with missing or invalid dates may introduce selection bias if IGE onset timing differs systematically between complete and incomplete records. Finally, follow-up data are unavailable; consequently we were unable to quantify how many patients with drug-related IGE subsequently developed pulmonary aspiration or other inhalational complications, and thus cannot estimate the proportion of IGE cases that progress to aspiration after drug exposure.

While causation cannot be inferred from spontaneous reports, our findings support several clinical precautions. Preoperative assessment should identify exposure to the nine drugs flagged in our model, particularly GLP-1 receptor agonists, with heightened awareness for female patients under 63 years. Adherence to existing GLP-1RA withholding guidelines is advised, and extended fasting or gastric ultrasound may be considered for other agents lacking formal recommendations. Future studies should prospectively measure gastric emptying, validate these signals in real-world clinical databases with adjustment for comorbidities, and assess whether the independent risk factors identified herein, namely, specific drugs, female sex, and age below 63 years, can be integrated into a risk stratification tool for drug-related IGE, which would require external validation prior to perioperative implementation.

## 5 Conclusion

In this real-world pharmacovigilance analysis, patients younger than 63 years, females, and users of any of nine specific drugs were found to have significantly higher reporting odds of drug-related IGE. These associations, however, do not establish causation. The signals observed for antidiabetic medications may be particularly confounded by the underlying severity and autonomic complications of diabetes mellitus, variables that are not captured in spontaneous reporting databases. In light of these inherent limitations, our findings should be viewed as hypothesis-generating signals that warrant confirmation in prospective studies designed to objectively measure gastric emptying and to adequately control for diabetic autonomic neuropathy. Nevertheless, our findings may provide practical reference for the timely recognition of medication-related IGE and for refining anaesthetic plans and perioperative patient management.

## Data Availability

The original contributions presented in the study are included in the article/Supplementary Material, further inquiries can be directed to the corresponding authors.
