# Supplementary material for: Risk factors for drug-related impaired gastric emptying: a pharmacovigilance analysis of the FDA adverse event reporting system
Source: Front Pharmacol. 2026 May 18;17:1844057. doi: 10.3389/fphar.2026.1844057 (PMC13223089; doi:10.3389/fphar.2026.1844057)

Supplementary Table S1. Two-by-two contingency table for disproportionality analysis.

| **Item** | **Target adverse events reported** | **Other adverse events reported** | **Total** |
| --- | --- | --- | --- |
| **Target drugs** | a | b | a + b |
| **Other drugs** | c | d | c + d |
| **Total** | a + c | b + d | a + b + c + d |

Supplementary Table S2. Four major algorithms used for signal detection.

| Method | Calculation formula | ﻿Criteria |
| --- | --- | --- |
| ROR | $ROR=\frac{a / c}{b / d}$ | a ≥ 3  95%CI (lower limit) > 1 |
|  | $SE(lnROR)=\sqrt{\frac{1}{a}+\frac{1}{b}+\frac{1}{c}+\frac{1}{d}}$ |  |
|  | $95\%CI= e^{\ln\left( ROR \right)\pm1.96se}$ |  |
| PRR | $PRR=\frac{a / (a+b)}{c / (c+d)}$ | a ≥ 3  PRR ≥ 2  $\chi2\geq4$ |
|  | $\chi2 =\frac{{(ad-bc)}^{2}(a+b+c+d)}{( a+b)(a+c)(c+d)(b+d)}$ |  |
| BCPNN | IC=${log}_{2}\frac{p(x,y)}{p(x)p(y)}={log}_{2}\frac{a(a+b+c+d)}{(a+b)(a+c)}$ | IC025>0 |
|  | E(IC)=${log}_{2}\frac{(a+\gamma11)(a+b+c+d+\alpha)(a+b+c+d+\beta)}{（a+b+c+d+\gamma）(a+b+\alpha1)(a+c+\beta1)}$ |  |
|  | $V\left( IC \right)=\frac{1}{{(ln2)}^{2}}\{\left[ \frac{\left( a+b+c+d \right)-a+\gamma-\gamma11}{\left( a+\gamma11 \right)\left( 1+a+b+c+d+\gamma\right)} \right]+\left[ \frac{\left( a+b+c+d \right)-\left( a+b \right)+\alpha-\alpha1}{\left( a+b+\alpha1 \right)\left( 1+a+b+c+d+\alpha\right)} \right]+\left[ \frac{\left( a+b+c+d \right)-\left( a+c \right)+\beta-\beta1}{\left( a+c+\beta1 \right)\left( 1+a+b+c+d+\beta\right)} \right]\}$ |  |
|  | $\gamma=\gamma11\frac{(a+b+c+d+\alpha)(a+b+c+d+\beta)}{(a+b+\alpha1)(a+c+\beta1)}$ |  |
|  | *IC-2SD=E(IC)-2*$\sqrt{V(IC)}$  $\alpha1=\beta1=1；\alpha=\beta=2；\gamma11=1$ |  |
| EBGM | $EBGM=\frac{a(a+b+c+d)}{\left( a+c \right)(a+b)}$ | EBGM05>2 |
|  | $SE(lnEBGM)=\sqrt{\frac{1}{a}+\frac{1}{b}+\frac{1}{c}+\frac{1}{d}}$ |  |
|  | $95\%CI= e^{\ln\left( EBGM \right)\pm1.96se}$ |  |

Equation: a, number of reports containing both the suspect drug and the suspect adverse drug reaction; b, number of reports containing the suspect adverse drug reaction with other medications (except the drug of interest); c, number of reports containing the suspect drug with other adverse drug reactions (except the event of interest); d, number of reports containing other medications and other adverse drug reactions. ROR, reporting odds ratio; CI, confidence interval; N, the number of co-occurrences; PRR, proportional reporting ratio; χ2, chi-squared; BCPNN, Bayesian confidence propagation neural network; IC, information component; IC025, the lower limit of the 95% one-sided CI of the IC; EBGM: Empirical Bayes Geometric Mean; EBGM05, The lower limit of the 90% one-sided CI of the EBGM.

Supplementary Table S3. The READUS-PV checklist

| **Section and topic** | **Item #** | **Checklist item** | **Location where item is reported** |
| --- | --- | --- | --- |
| **Title** |  |  |  |
|  | *1a* | If disproportionality analyses are a prominent component of the published study, the study should be identified as a “disproportionality analysis”. The type of data and name of the database(s) should be specified. | Page 1 |
|  | *1b* | Report the name of adverse event(s) and/or drug(s) under study, when applicable. | Page 1 |
| **Introduction** |  |  |  |
| Background | *2a* | Describe the drug(s) and its utilization, the nature of the adverse event(s) under study and its frequency, and the existing knowledge on the drug-event combination. | Page 2-3 |
|  | *2b* | Specify the rationale for performing the analysis, e.g., as part of routine pharmacovigilance, to investigate an overall safety profile, or to assess a pre-specified hypothesis. | Page 2-3 |
|  | *2c* | Explain why ICSR databases and disproportionality analysis are suitable to fill the knowledge gap. | Page 3 |
| Objectives | *3* | State specific objectives, identifying the adverse event(s), the drug(s), and the reference group, including any pre-specified hypothesis, if applicable. | Page 3 |
| **Methods** |  |  |  |
| Study design | *4a* | Identify the study (i.e., “disproportionality analysis”) and the type of data used (e.g., “individual case safety reports”). | Page 4 |
|  | *4b* | Provide an outline of the entire study design, including primary and sensitivity analyses performed, and other designs such as case-by-case analysis or literature review. | Page 4-5 |
| Data description, access, and pre-processing | *5a* | Specify the name of the database(s), the database(s) custodian, and the coverage. Specify the type/number of drugs included within the database and the thesaurus, taxonomies, or ontologies used for coding drugs and events. | Page 4-5 |
|  | *5b* | Specify the extraction dates and describe and justify all choices used for data pre-processing, including any data transformation or exclusion, if appropriate. | Page 4-5 |
| Variables definition | *6a* | Describe the study population, including any restriction. | Page 4-5 |
|  | *6b* | Describe the nature and the meaning of key variables assessed in the work. | Page 4-5 |
|  | *6c* | Specify and justify any grouping of drugs or events. For drugs, specify and justify whether active ingredients/trade names/salts were considered and/or the selected role. | Page 5 |
|  | *6d* | Describe any additional data source used, the type of data, and how they interact with ICSRs. | Not applicable |
| Statistical methods | *7a* | Present any descriptive analysis performed, specifying variables investigated, statistical tests, and significance thresholds. | Page 4-6 |
|  | *7b* | Describe the measure(s) selected for the disproportionality analysis including any threshold used to identify signals of disproportionate reporting. Explain the reason for this choice if applicable. | Page 4-6 |
|  | *7c* | Clearly describe any sensitivity analysis and any tool to control confounding, including any restriction, subgroup, stratification, adjustment, or interaction. | Page 4-6 |
|  | *7d* | Specify the variables and methods used for the case-by-case analysis, including any algorithm or criteria used to assess causality, if performed. | Not applicable |
|  | *7e* | Specify any statistical methods used for other data sources. | Not applicable |
| **Results** |  |  |  |
| Participants | *8a* | Specify the number of individual case safety reports included at each stage, including reasons for exclusion. | Page 6 |
|  | *8b* | Provide key demographic and clinical characteristics of cases, if possible comparing cases with any appropriate reference group. | Page 6 |
| Disproportionality analysis | *9* | Present all results including confidence intervals. Present also results of sensitivity analyses, if performed. | Page 7 |
| Case-by-case analysis | *10* | Present the case-by-case analysis of key variables. Present the causality assessment, if applicable. | Page 7-8 |
| **Discussion** |  |  |  |
| Key results | *11* | Discuss key results with reference to study objectives and contextualize them within the current literature and other consulted sources. Clearly discriminate between expected reactions and emerging safety signals. | Page 8-9 |
| External validity | *12a* | Discuss the external validity of the results to the general population. | Page 9-11 |
|  | *12b* | Discuss the potential relevance of results in clinical practice | Page 9-1 |
|  | *12c* | Propose further study designs if applicable | Not applicable |
| Limitations | *13* | Present general limitations, making clear that disproportionality analysis alone cannot prove causation or measure incidence, and specific limitations, including confounding and reporting bias and efforts to mitigate them. | Page 11 |
| **Declarations** |  |  |  |
|  | *14a* | Provide the source of funding/sponsorship and the role of the funders/sponsors for the present study and for any original study on which the present article is based. | Page 12 |
|  | *14b* | Clearly identify potential commercial and intellectual conflicts of interest (e.g., link to any drug/event investigated, whether financial, legal action, or software used). | Page 12 |
|  | *14c* | Declare any institutional approval needed or granted in the investigation. | Page 12 |
|  | *14d* | Include a statement on data availability, code availability (including the version of the statistical software used), and protocol registration. | Page 13 |

Supplementary Table S4. Signal detection of pulmonary aspiration related to IGE in the top 10 drugs within FAERS.

| **Drug** | **pulmonary aspiration cases/IGE cases** | **ROR (95% CI)** | **PRR (χ^2^)** | **EBGM (EBGM05)** | **IC (IC025)** |
| --- | --- | --- | --- | --- | --- |
| Semaglutide | 49/815 | 2.32 (1.75 - 3.07) | 2.32 (36.54) | 2.31 (1.75) | 1.21 ( 0.76 ) |
| Dulaglutide | 12/601 | 0.34 (0.19 - 0.6) | 0.34 (15.43) | 0.34 (0.19) | -1.56 ( -2.28 ) |
| Tirzepatide | 14/559 | 0.33 (0.2 - 0.56) | 0.33 (18.61) | 0.33 (0.2) | -1.58 ( -2.26 ) |
| Calcium chloride/glucose/magnesium chloride/sodium chloride/sodium lactate | 77/328 | 1.07 (0.86 - 1.34) | 1.07 (0.35) | 1.07 (0.85) | 0.1 ( -0.23 ) |
| Oxybate sodium | 52/197 | 1.6 (1.22 - 2.1) | 1.6 (11.58) | 1.59 (1.21) | 0.67 ( 0.26 ) |
| Exenatide | 10/190 | 0.26 (0.14 - 0.48) | 0.26 (21.59) | 0.26 (0.14) | -1.96 ( -2.73 ) |
| Liraglutide | 4/162 | 0.24 (0.09 - 0.63) | 0.24 (9.94) | 0.24 (0.09) | -2.08 ( -3.14 ) |
| Insulin lispro | 19/138 | 0.46 (0.29 - 0.72) | 0.46 (11.99) | 0.46 (0.29) | -1.12 ( -1.72 ) |
| Olmesartan | 2/124 | 0.28 (0.07 - 1.11) | 0.28 (3.74) | 0.28 (0.07) | -1.84 ( -3.11 ) |
| Esomeprazole | 109/108 | 3.05 (2.53 - 3.69) | 3.05 (148.68) | 3.03 (2.51) | 1.6 ( 1.3 ) |

IGE, impaired gastric empty; FAERS, Food and Drug Administration Adverse Event Reporting System; ROR, reporting odds ratio; CI, confidence interval; PRR, proportional reporting ratio; χ2, chi-squared; BCPNN, Bayesian confidence propagation neural network; IC, information component; IC025, the lower limit of the 95% one-sided CI of the IC; EBGM: Empirical Bayes Geometric Mean; EBGM05, The lower limit of the 90% one-sided CI of the EBGM.

Supplementary Figure S1. Violin plot of time to drug-related impaired gastric emptying occurrence.


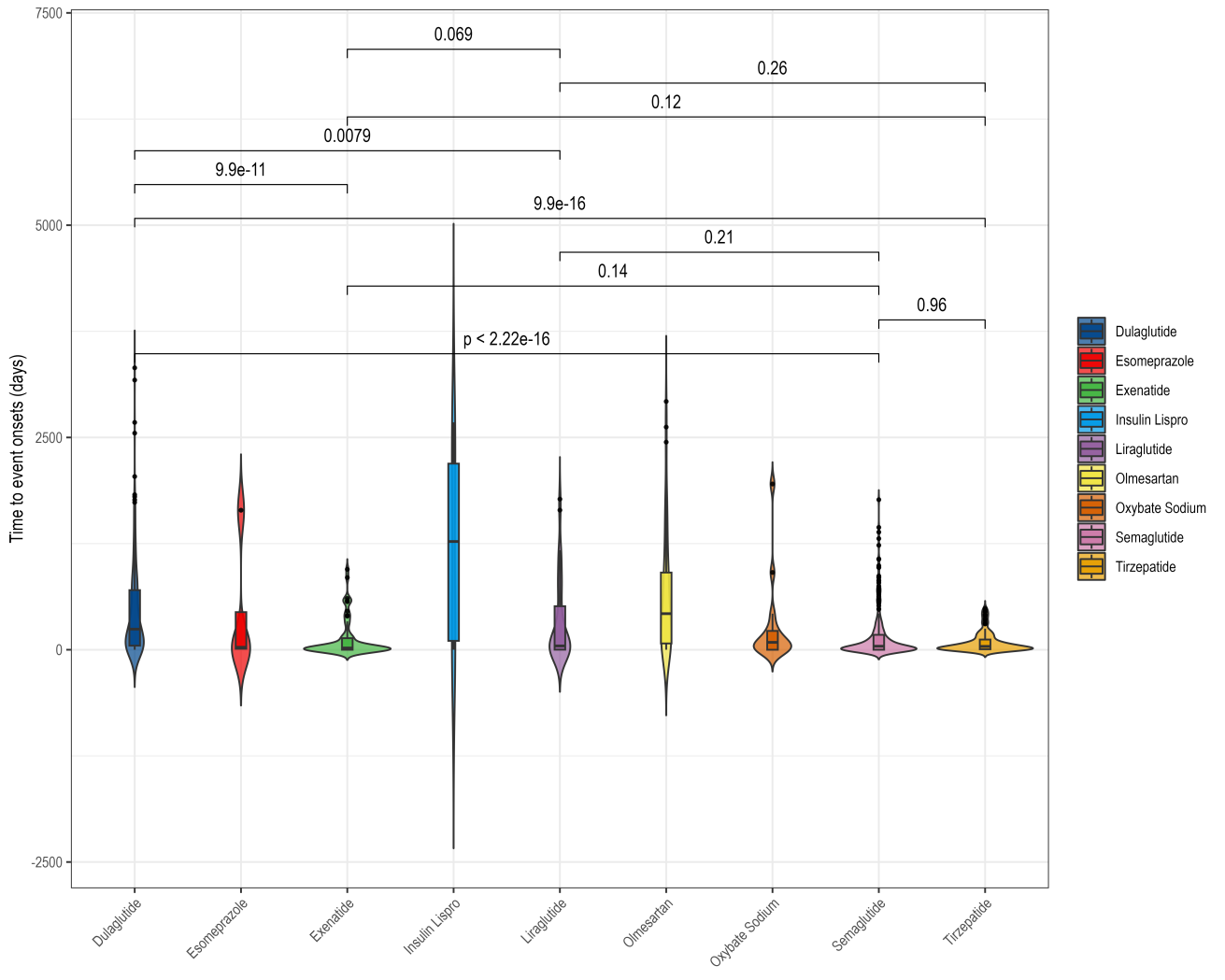

Supplement: Supplementary file 1 [file Supplementaryfile1.docx]
